# Exploring the Prospects of Macadamia Nutshells for Bio-Synthetic Polymer Composites: A Review

**DOI:** 10.3390/polym15194007

**Published:** 2023-10-06

**Authors:** Md Sefat Khan, Md Mainul Islam, Jayantha Epaarachchi, Shinichi Shibata

**Affiliations:** 1 Centre for Future Materials and School of Engineering, University of Southern Queensland, Toowoomba, QL 4350, Australia; 2 Department of Mechanical Systems Engineering, University of the Ryukyus, Okinawa 903-0213, Japan

**Keywords:** macadamia nutshells, polymer composites, mechanical characterizations, morphology

## Abstract

The global production of macadamia nuts has witnessed a significant increase, resulting in the accumulation of large quantities of discarded nutshells. These nutshells possess the properties of remarkable hardness and toughness, which are comparable to those of aluminum. Incorporating natural fillers to enhance the properties of composite materials for various applications, including light duty, structural, and semi-structural purposes, is a common practice. Given their inherent hardness and toughness, macadamia nutshells present an intriguing choice as fillers, provided that the manufacturing conditions are economically viable. With the urgent need to shift toward natural fillers and reduce reliance on synthetics, exploring macadamia nutshells as components of natural fiber composites becomes imperative. This review aims to comprehensively examine the existing body of knowledge on macadamia nutshells and their bio-synthetic polymer composites, highlighting key research findings, achievements, and identifying knowledge gaps. Furthermore, the article will outline prospective areas of focus for future research endeavors in this domain, aligning with the universal goal of minimizing synthetic materials.

## 1. Introduction

In recent times, growing concerns regarding environmental issues, health considerations, and the urgent need for sustainable manufacturing methods have amplified the focus on the research on and development of green materials for engineering applications. Natural fibers derived from sources such as bamboo, flax, coir, hemp, etc., have been employed as reinforcements in the production of bio-composites [1]. Effective waste management has become imperative to maintaining a livable environment, as agricultural activities generate substantial byproducts that often end up in landfills. Macadamia nutshells, like other agricultural residues, are commonly incinerated as solid biomass fuel or utilized as organic fertilizer, cooking fuel, garden mulch, and animal bedding, or discarded altogether. Notably, macadamia nutshells exhibit distinct structural and density characteristics compared to those of natural wood, although their chemical composition is surprisingly similar [2]. Macadamia, a genus within the Proteaceae family, encompasses several species primarily found in Australia. While ten species have been identified, only two are grown commercially in Australia*, Macadamia integrifolia* and *Macadamia tetrophylla.* The former is preferred due to its higher oil content and improved nut edibility after roasting, making it the dominant species for commercial plantations [3]. In Australia alone, the food industry generates approximately 28,000 tons of empty macadamia shells as a byproduct [4]. The global market for macadamia nuts continues to expand, with shells and other waste comprising nearly 70% of the fruit’s weight [5]. Unfortunately, the potential of macadamia shells as byproducts remains significantly underutilized [6]. As a botanical structure, macadamia nutshells are delicately optimized, exhibiting exceptional strength and toughness resulting from ecological evolution and natural selection. These nutshells possess a cellular solid mass with relatively high strength and low density; however, their structure differs considerably from that of trees, displaying isotropic and uniform characteristics [7]. Macadamia nutshell-filled polymeric composites hold promising potential for manufacturing various structural components, including sandwich composites, with prospective applications in infrastructure, aerospace, and automotive industries. Additionally, macadamia shells can be processed at high temperatures in a specialized low-oxygen environment to produce biochar, which finds applications in carbon filters, life-saving medical treatments, industrial nano-powders, and cosmetics.

Although research on macadamia nutshell-reinforced composites is limited, no comprehensive review has been published thus far. Therefore, this article aims to provide an overview of bio-synthetic polymer composites utilizing macadamia nutshells, encompassing their chemical properties, mechanical characteristics, manufacturing methods, fire retardancy, water absorption behavior, and thermal properties.

## 2. Bio-Synthetic Polymer Composites

**Figure 1:**
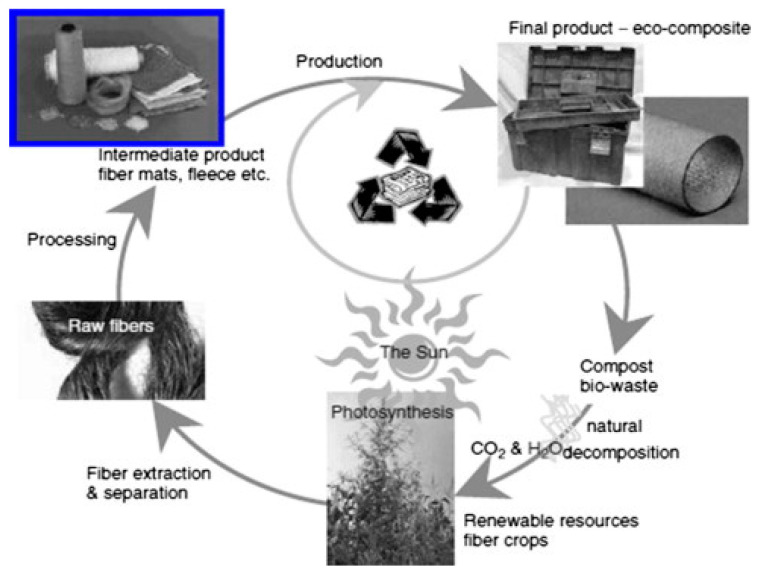
Life cycle of bio-synthetic polymer composites. Reprinted with permission from Ref. [12]. Copyright 2011, Elsevier.

Researchers have long been exploring opportunities to incorporate natural fibers into various industries, a trend that gained momentum with the emergence of polymers in the early 19th century. Synthetic polymers and composites have become ubiquitous worldwide; however, their production and recycling processes contribute to environmental pollution, prompting the need for alternative solutions utilizing natural fibers. Bio-synthetic composites, composed of natural materials and artificially prepared or synthesized polymers, offer strength and structural integrity to the final product. Cellulosic fibers derived from sources such as flax, alpaca, hemp, jute, and wood are biodegradable and commonly used as reinforcement in composites with different thermoplastic matrices. Bio-composite materials offer numerous advantages over conventional materials, including higher specific strength, stiffness, and fatigue resistance, thereby enabling more adaptable structural design. Additionally, bio-composites are biodegradable, possess superior tensile strength, exhibit low specific gravity, and are recyclable. Consequently, these materials find application in diverse product manufacturing and innovative fields. Green composites, compared to their synthetic counterparts, offer various advantages, including reduced tool wear [8] and biodegradability [9]. Furthermore, natural fibers exhibit higher specific strength than do glass fibers while maintaining a similar specific modulus [10]. Many of these fibers are obtained through the processing of agricultural, industrial, or consumer waste [11]. Natural fibers are widely accessible and find applications in a wide range of industries, as illustrated in Figure 1.

## 3. Macadamia Nutshells

Nutshells, which are agricultural waste, consist of lignocellulosic materials. The strength and stiffness of agro-fibers are primarily attributed to cellulose content. Traditionally, nutshells have been limited to low-end applications. However, recent research has focused on exploring enhanced and value-added uses for these nutshells. Some potential applications include utilizing them as carriers for insecticides and pesticides, employing them as abrasives for cleaning and polishing purposes, utilizing them as thickeners in paint formulations, and utilizing them as raw materials for activated carbon production. Moreover, in recent years, researchers have extensively investigated the potential of incorporating nutshells as fillers in polymer composites [3].

**Figure 2:**
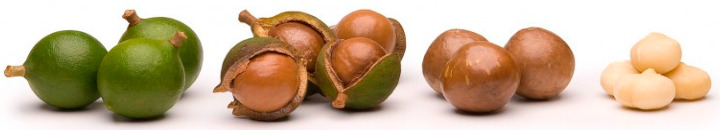
Macadamia nuts. Adapted with permission from Ref. [14]. Copyright 2014, Schuler et al.

Macadamia nutshells offer a promising opportunity to develop innovative green composites via the incorporation of them as fillers with compatible polymeric resins. Nutshells fall into the category of three-dimensional cells, characterized by cell walls with a random orientation. From the early stages after flowering, the nuts develop and remain within the shells until they reach their final lifecycle stage (Figure 2). Macadamia trees thrive in sub-tropical climates and are long-term crops, typically taking four to five years from planting to reach the point of cropping. Fully commercially viable yields are obtained within approximately seven years, with an annual growing cycle of around 9 months. The first flowering typically occurs in early spring, followed by the development of small nutlets. These nutlets grow and mature during spring and summer, ripening in early autumn. Each cluster of 40–50 flowers produces 2–15 nutlets, resulting in large clusters of plump green nuts by early autumn. Harvesting usually begins in late autumn and continues throughout the winter months. Farmers collect the nuts once they have fallen to the ground, conducting harvest rounds every two to four weeks. After removing the soft outer husk, the nuts are transferred to drying facilities, where they undergo controlled temperature and humidity conditions to reduce their moisture content from 15–20% to 3.5%—a crucial step before cracking [13]. Careful mechanical cracking is employed to minimize damage to the delicate kernel, and the waste shells and kernels are subsequently separated. The germination process of macadamia seeds covered by shells occurs in five stages, taking approximately 15–115 days from sowing. The prolonged germination period can be attributed to the hard and rigid structure of the shells.

**Figure 3:**
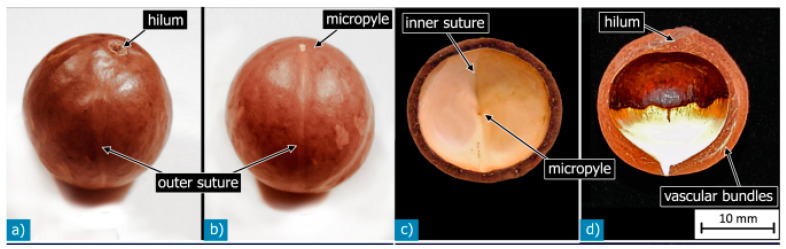
Botanical nomenclature of macadamia nutshell. (**a**,**b**) Outer layer; (**c**,**d**) Innermost layer. Reprinted with permission from Ref. [14]. Copyright 2014, Schuler et al.

**Figure 4:**
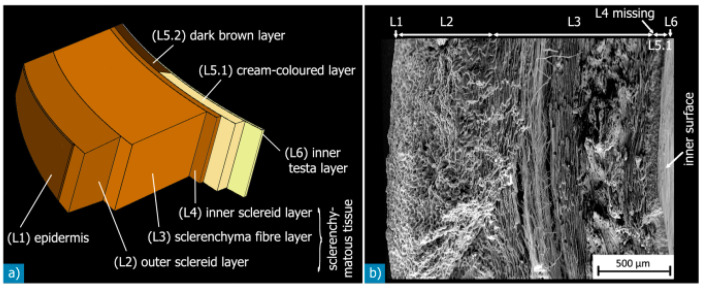
Complex structures of macadamia nutshell. (**a**) Schematic illustration of these layers; (**b**) SEM image of a cross section where layers are visible and have been classified. Reprinted with permission from Ref. [14]. Copyright 2014, Schuler et al.

**Figure 5:**
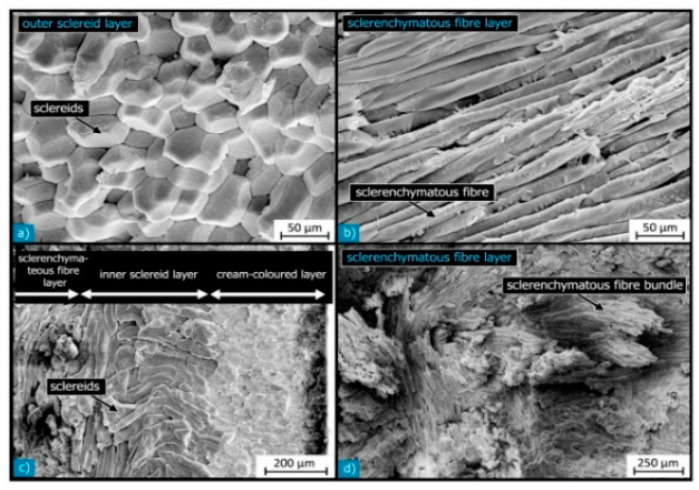
SEM images of layers of cellulosic fibers. (**a**) Sclereid layer; (**b**) Sclerenchymatous layer; (**c**) inner sclereid layer (L4); (**d**) sample of fibrous layers in compact bundles. Reprinted with permission from Ref. [14]. Copyright 2014, Schuler et al.

**Figure 6:**
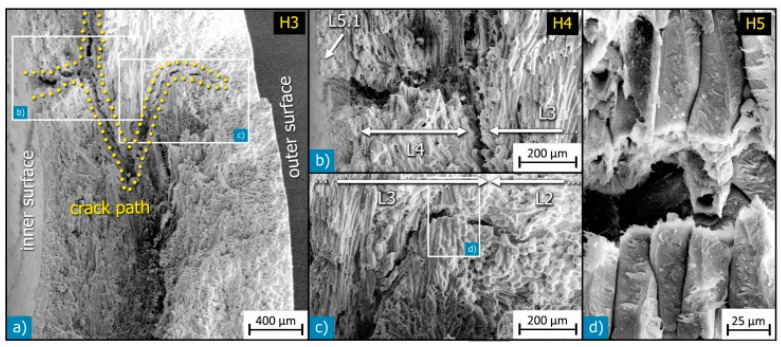
SEM images on Crack propagation in macadamia seed coat. (**a**) Compressed loading on samples; (**b**) Enlarged view showing interfacial fracture deflection between sclerenchymatous layer (L3) and sclereid layer (L4); (**c**,**d**) Ending of crack. Reprinted with permission from Ref. [14]. Copyright 2014, Schuler et al.

Macadamia nutshells possess various anatomical features, including the hilum, micropyle, outer suture, inner suture, vascular bundles, sclerenchyma fiber layer, sclereid, and testa layers (Figure 3). These terms belong to the realm of botanical study, and their understanding is relevant in the context of material science and mechanical perspectives. Figure 4 showcases a diagram illustrating the sandwich structure of a typical macadamia seed shell, accompanied by a scanning electron microscope (SEM) image that highlights the dense arrangement of the shell layers. Figure 5 provides detailed views of each individual layer within the shell structure. Additionally, Figure 6 illustrates the development of cracks in the shells under various loading conditions.

## 4. Chemical Properties

In a study investigating the production of composite panels using macadamia nutshells and waste plastic from the automotive industry, the researchers conducted an analysis to assess the elemental content, moisture content, volatile carbon, and fixed carbon of the nutshells. This analysis involved the use of X-ray fluorescence (XRF) spectroscopy, along with proximate analysis (as presented in Table 1), ultimate analysis, and elemental analysis (as presented in Table 2) [15].

**Figure 7:**
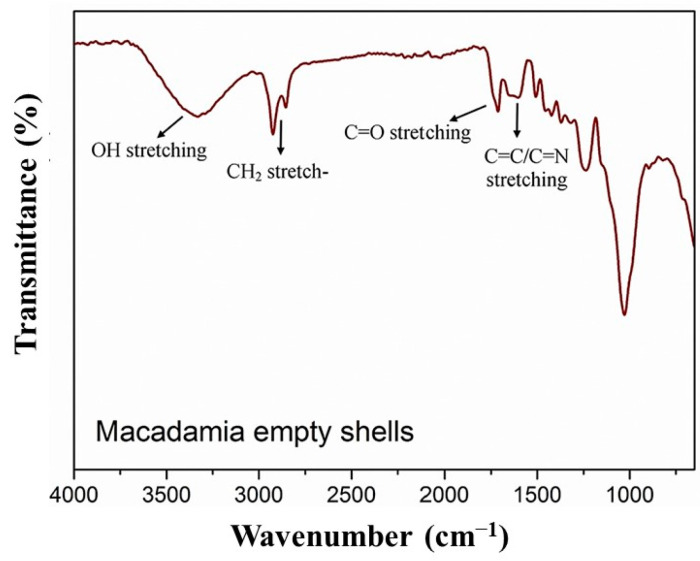
FTIR spectrum of neat macadamia empty shells. Reprinted with permission from Ref. [15]. Copyright 2017, Elsevier.

The chemical structure of macadamia nutshells was evaluated through Fourier transform infrared spectroscopy (FTIR) analysis, which revealed the presence of numerous functional groups in the FTIR spectrum (Figure 7). The shells predominantly consist of polymers such as cellulose, hemicellulose, and lignin. The characteristic peaks corresponding to OH, CH_2_, C=O, C=C, and C-O-C functional groups provide evidence of the presence of cellulose, hemicellulose, and lignin structures within macadamia nutshells.

Notably, the peaks observed at 2910 and 2930 cm^−1^ indicate C-H stretching, while the peak in the region of 1720 cm^−1^ may be attributed to the presence of acetyl groups in hemicellulose. Peaks around 1600 and 1510 cm^−1^ suggest the presence of aromatic structures, and the strong peak at 1000 cm^−1^ corresponds to stretching vibrations from the functional groups present in hemicellulose and cellulose structures.

## 5. Thermal Analysis

**Figure 8:**
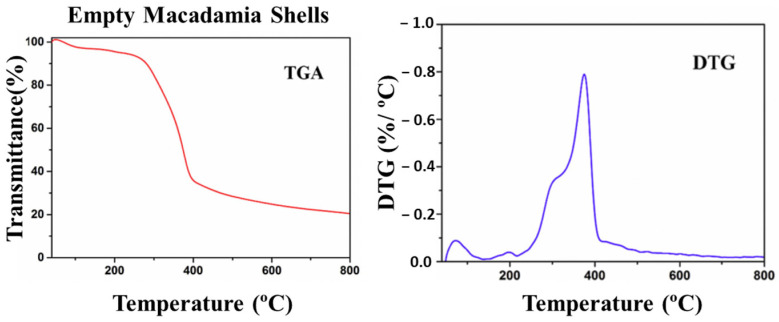
TGA and DTG curve of automotive waste plastic and empty macadamia shells. Reprinted with permission from Ref. [15]. Copyright 2017, Elsevier.

To investigate the thermal behavior of macadamia nutshells, Sagar et al. [15] performed thermo-gravimetric analysis (TGA), derivative thermo-gravimetric (DTG) analysis, and pyrolysis experiments on empty nutshells (Figure 8). TGA tests involved measuring the mass of the samples as a function of temperature. During TGA, changes in mass occur due to sublimation, evaporation, decomposition, chemical reactions, and magnetic or electrical transformations, providing insights into the thermal stability of the material. The presence of acetyl groups makes hemicellulose the least thermally stable component, leading to its decomposition at around 300 °C, as observed via the initial weight loss. Cellulose degradation occurs rapidly around 400 °C, indicated by a characteristic peak on the DTG curve (at approximately 370 °C). Lignin degradation, on the other hand, takes place at lower temperatures and spans a wider temperature range. At the end of the TGA analysis, the macadamia nutshells left behind a residue of approximately 20%.

The thermal analysis results confirm that macadamia nutshells exhibit thermal stability up to 250 °C. Furthermore, the TGA results provide confirmation of the presence of cellulose (~35%), hemicellulose (~30%), and lignin (~35%) in macadamia nutshells.

## 6. Mechanical Properties

The usability of any material in terms of engineering applications highly depends upon its mechanical attributes, such as flexural properties, tensile properties, fracture toughness, stress and strain curves. These characteristics mostly determine the probable behavior of a material in a specified application. Unlike man-made composites, biological materials do not rely on simply one fiber–matrix interface, but rather on interactions of the multiplicity of interfaces between the different structural levels of molecules, cells, and fibers. Studies have been conducted to determine the mechanical characteristics of macadamia nutshells, such as flexural properties, tensile properties, hardness, and compressive strength. They are known to have approximately the same fracture toughness as that of common ceramics and glass; when compared based on specific strength or modulus, they exceed those of these materials due to their low density.

Wang, Zhang and Mai [13] reported that the elastic modulus and strength of macadamia nutshells is around 5.2 GPa and 40–50 MPa, respectively. Mechanical properties of macadamia nutshells subjected to heat treatment were studied. Researchers performed elastic stress analysis under diametrical compression and observed that cracks caused the final fracture, initiated from the inner surface of the shell underneath the loading point. Representatives were collected as green, dried, and boiled. C-ring specimens were prepared from whole nuts to conduct mechanical tests. Three geophysical positions were considered such as the north pole, south pole, and equator for the specimens (Figure 1 of reference [14]).

It is shown from Table 3 and Table 4 that macadamia nutshells have a high compressive strength, which makes it hard to prevent insects from boring into the nuts. According to the study, their density is between 1.2 × 10^3^ and 1.5 × 10^3^ kg/m^3^, and macadamia nutshells are heavier than most hard and soft woods.

**Figure 9:**
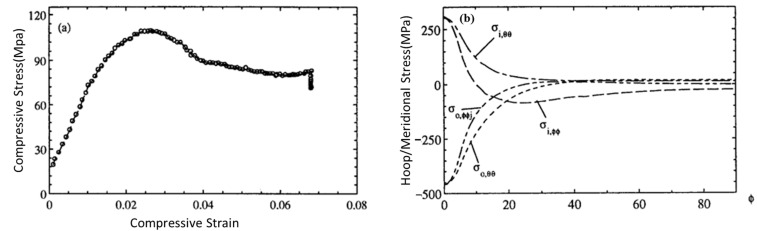
(**a**) Stress–strain curve and (**b**) Elastic stress distributions of macadamia nutshell. Adapted with permission from Ref. [13]. Copyright 1995, Kluwer Academic Publishers.

The stress–strain curve and elastic stress distribution curve were evaluated (Figure 9). Stresses in the shell were designated as meridian and hoop stresses adopting a geophysical analogy by Jennings et al. [16].

## 7. Macadamia Nutshell Reinforced Composites

The unique characteristics of macadamia nutshells, such as their low density, high mechanical strength [17,18], biodegradability, and recyclability, make them highly suitable for a wide range of innovative product designs. Researchers have extensively studied the performance of composites composed of macadamia nutshells, pine cone wastes [19] and various polymers, including poly lactic acid (PLA) [20], polyethylene (PE), polyester, polybenzoxazine [21], polypropylene (PP) [22], as well as certain resins [23,24,25]. 

### 7.1. Composites with Poly Lactic Acid

Chensong et al. [17] conducted a study on the mechanical properties of bio-composites composed of macadamia nutshell powder and poly lactic acid (PLA). The strength and stiffness of the composites were found to be dependent on the weight content of macadamia nutshell particles. Specifically, a 40% weight content of nutshell powder resulted in a 9.8% increase in the elastic modulus. However, the hardness of the composites was not affected by the weight content, and an increase in powder content led to a decrease in both flexural and tensile strength. Rakesh et al. [26] investigated composites of PLA with the addition of a plasticizer, such as Triacetin. The inclusion of the plasticizer caused changes in the morphology of the composites. Among the tested compositions, the composite with 8% plasticizer exhibited a maximum tensile strength of approximately 11 MPa, along with satisfactory elongation at break.

**Figure 10:**
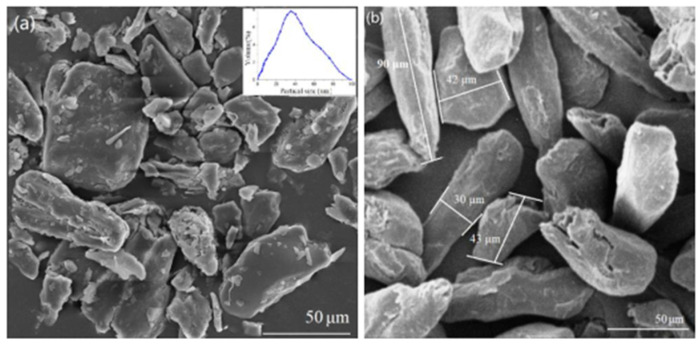
Morphology and particle distribution of macadamia nutshell particles: (**a**) Before treatment and (**b**) After treatment with NaOH/silane. Reprinted with permission from Ref. [27]. Copyright 2020, Xiaohui et al.

**Figure 11:**
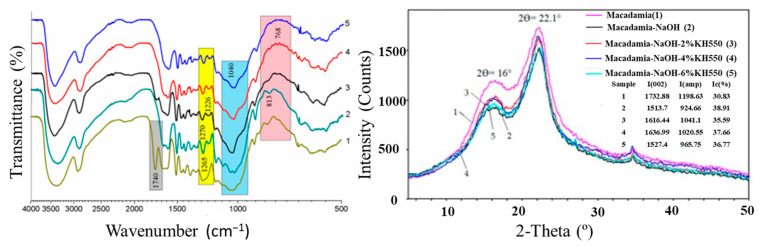
The FTIR spectra of representatives: (1) MS; (2) NaOH-treated MS; (3) NaOH + 2 wt% KH550-treated MS; (4) NaOH + 4 wt% KH550-treated MS; (5) NaOH + 6 wt% KH550-treated MS. Reprinted/adapted with permission from Ref. [27]. Copyright 2020, Xiaohui et al.

In a study by Xiaohui et al. [27], the additive manufacturing of composites using poly lactic acid (PLA) and macadamia nutshell (MS) was explored. The macadamia shell samples were treated with alkali and silane, resulting in morphological changes. The PLA composite containing 10 wt% of the treated macadamia nutshell showed thermal and mechanical properties comparable to those of pure PLA, as well as promising characteristics for scaffold applications. Morphological changes resulting from the treatments are depicted in Figure 10, while Figure 11 illustrates the FTIR spectra and XRD analysis of the treated and untreated macadamia shell, demonstrating changes in chemical bonds and crystallinity. The PLA composite with 10% treated macadamia nutshell exhibited the best performance, showing potential for use in lightweight and structural parts.

In another report, the authors studied such composites made of four kind of nutshells such as walnut, almond, macadamia (MSP) and wild almond [28]. The PLA/MSP composite was the most water-resistant regardless of surface treatment. Crystallization degree of these representatives also improved.

### 7.2. Composites with Polyethylene and Polyester

Sevda et al. [29] conducted a study on high-density polyethylene (HDPE) composites reinforced with microcrystalline cellulose (MCC) and nutshell fiber (N). The researchers also incorporated polyethylene graft maleic anhydride (PE-g-MA) to enhance the interface between the components. The prepared samples were subjected to accelerated weathering for 672 h in total, during which changes in morphology, weathering, mechanical properties, and chemical composition were analyzed.

**Figure 12:**
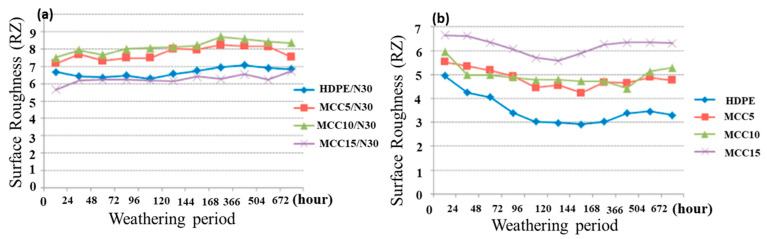
Surface roughness of composite representatives: (**a**) Nutshell/ MCC-reinforced composites and (**b**) MCC-reinforced composites along with weathering period. Adapted with permission from Ref. [29]. Copyright 2021, Sevda et al.

Exposure to weathering conditions led to a decrease in flexural strength and an increase in the modulus of elasticity of 62%. Color changes and a loss of gloss were predominantly observed in the MCC/nutshell reinforced composites, along with an increase in surface roughness. Figure 12 depicts the surface roughness patterns of eight different composite samples subjected to varying weathering periods [29].

Laert et al. [7] conducted a study on the mechanical and thermal properties of low-density polyethylene (LDPE) composites incorporating Macadamia integrifolia residue. Various fiber contents (0%, 5%, 10%, and 20% by weight) were investigated, and it was found that the composites with a 20% fiber content performed the best. The inclusion of fibers increased the stiffness of the composites compared to neat LDPE, but this led to a reduction in toughness and resilience, resulting in lower impact energy absorption. Chensong et al. [30] investigated the flexural properties of polyester composites reinforced with macadamia nutshell particles at four weight fractions: 10%, 20%, 30%, and 40%. The presence of voids in the composites was observed to decrease the flexural strength. The authors also reported that the flexural strength of the polyester did not improve with the addition of macadamia nutshell particles.

### 7.3. Composites with Polypropylene (PP)

**Figure 13:**
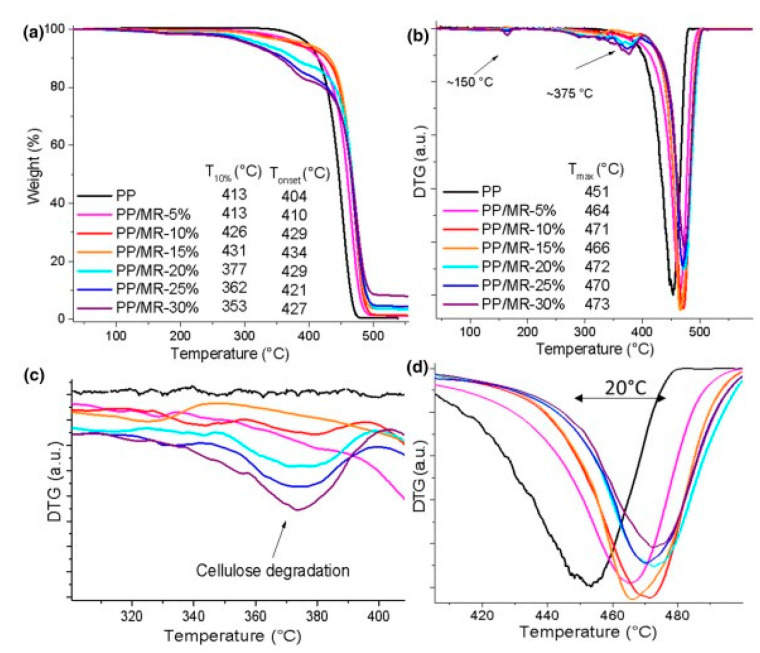
Thermogravimetric curves of pure PP and MR composites in several ratios. (**a**) The TGA graphs of pristine PP and its composites; (**b**) Respective DTGS; (**c**,**d**) Cellulose degradation pattern. Adapted with permission from Ref. [31]. Copyright 2021, Lucas et al.

Lucas et al. [31] studied composites made of macadamia nutshell residues (MR) and polypropylene (PP) composites using different MR contents (5, 10, 15, 20, 25, and 30%wt). Characterizations were conducted mainly focusing on the effect of moisture retention, and additionally, life cycle assessment (LCA) was obtained. The presence of MR content allows thermal stability. Meanwhile, it creates cracks and voids in the interface although it does not affect mechanical performance substantially (Figure 13). TGA and DTG curves, and the nature of moisture retention during the 7 days of the representatives were obtained. LCA revealed higher MR contents (30%) to promote lower environmental impacts than does the classical handling of nutshells (Figures 7 and 8 of reference no. [31]).

Nycolle et al. [32] investigated the effect of an alkaline treatment and coupling agent on the thermal and mechanical properties of macadamia nutshell residue (5 to 30% wt)-based PP composites. Such a treatment allows interfacial adhesion between the fiber and matrix. The FTIR spectra and X-ray diffraction pattern (XRD) obtained from the representatives before and after the treatment present how effectively functional groups were changed and crystallinity was transformed. Thermal degradation and mechanical properties were studied along with morphological analysis to observe the performance of treatments of the fibers and how they interfaced with the matrix. An addition of 30% wt treated fiber to the PP exhibited an enhancement of 67.5% in the tensile modulus. However, it was established that a higher fiber content being added to the PP enhanced the stiffness, and consequently reduced the impact strength of the materials (Figures 2, 4 and 7 from reference [32]).

### 7.4. Composites with Some Resins

**Figure 14:**
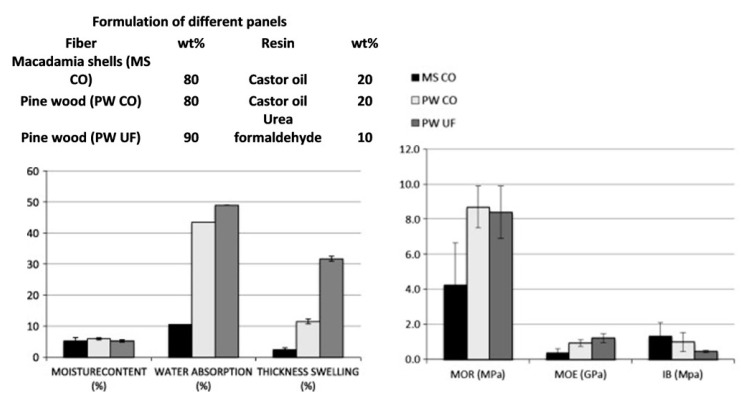
Moisture retention; strength behavior (modulus of rupture, modulus of elasticity and internal bond strength) of particleboard samples. Reprinted/adapted with permission from Ref. [33]. Copyright 2013, Elsevier.

Wechsler et al. [33] conducted a study comparing particleboards made from macadamia nutshells with resin derived from castor oil to conventional wood fiber/urea formaldehyde particleboards. The macadamia nutshell particleboards exhibited a 43% higher density, lower moisture retention, and reduced swelling. The internal bond strength was similar, but the modulus of rupture and modulus of elasticity were slightly lower compared to those of the conventional particleboards. Figure 14 presents the relevant data recorded in the study. Furthermore, the particleboards made with castor oil resin emitted less than 5% formaldehyde compared to traditional urea formaldehyde particleboards.

Derrick et al. [34] investigated the physico-mechanical properties of composite particleboards made from macadamia nutshells and Gum Arabic. The samples containing 50% Gum Arabic and 50% macadamia nutshells demonstrated favorable results, including the lowest average values of water absorption and swelling after submersion in distilled water, as well as the highest density, modulus of rupture, modulus of elasticity, internal bond strength, and compressive strength. A comprehensive study was conducted to determine the influence on the physico-mechanical properties of particleboards fabricated with particles of *Eucalyptus saligna* and macadamia nutshells [35]. Urea formaldehyde (UF)-based resin, an ammonium sulfate catalyst, and a paraffin emulsion were used in the fabrication process. The results indicated that particleboards with a high proportion of macadamia nutshell particles exhibited lower mechanical strength and dimensional stability. This was attributed to the thicker geometry of the macadamia nutshell particles, which limited their interaction with the adhesive.

**Figure 15:**
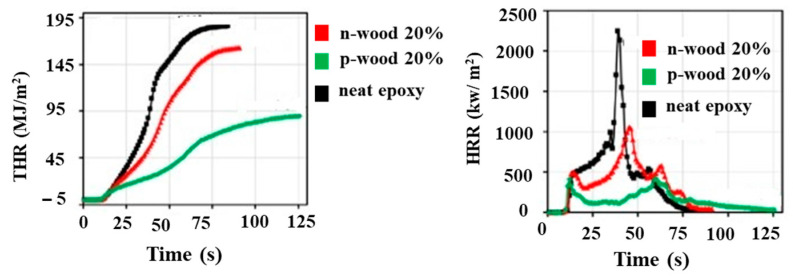
Heat release characteristics of specially treated bio composites. Adapted with permission from Ref. [36]. Copyright 2021, American Chemical Society.

Omid et al. [36] also investigated the use of synthesized phosphorous-based deep eutectic solvents or phosphorylated macadamia nutshell powder (p-wood) as a reinforcement in epoxy resin composites. The composites containing 20% p-wood exhibited a V-1 rating in the UL 94 Classification of the plastics flammability standard, along with significant reductions of 74% in the peak heat release rate and 344°C in the maximum smoke temperature compared to those of neat epoxy resin. The composites also showed an increase in char yield and limiting oxygen index value. Figure 15 illustrates the heat release rate (HRR) and total heat release (THR) rate of the bio-composite samples, where “N-wood” refers to macadamia nutshell powder treated with a toluene solvent.

A study focused on the thermal behavior of benzoxazine composites reinforced with macadamia biomass was conducted [37]. Differential scanning calorimetry (DSC), dynamic mechanical analysis (DMA), and thermomechanical analysis (TMA) were employed to investigate the properties of the composites. The results showed that the addition of 10% (*v*/*v*) of macadamia biomass in its natural form had minimal impact on the glass transition temperature, modulus of elasticity, and linear thermal expansion coefficient of the benzoxazine matrix.

### 7.5. Composites from Waste Macadamia Nutshells and Automotive Waste Plastic

A study focused on the production of wood plastic composite (WPC) panels via a combination of waste automotive plastics with macadamia nutshells as a matrix material was conducted [15]. The study examined the density, mechanical properties, microstructure, and thermal properties of the produced WPC panels and compared them with those of panels made solely from 100% automotive plastic. The investigation revealed that the addition of macadamia shells to the automotive waste plastic helped improve the modulus of elasticity under compression loading. The comprehensive modulus for the automotive waste plastic was 253 MPa. Since wood has a higher modulus than plastic does, the modulus of the fabricated panels increased with an increase in the content of macadamia shells.

**Figure 16:**
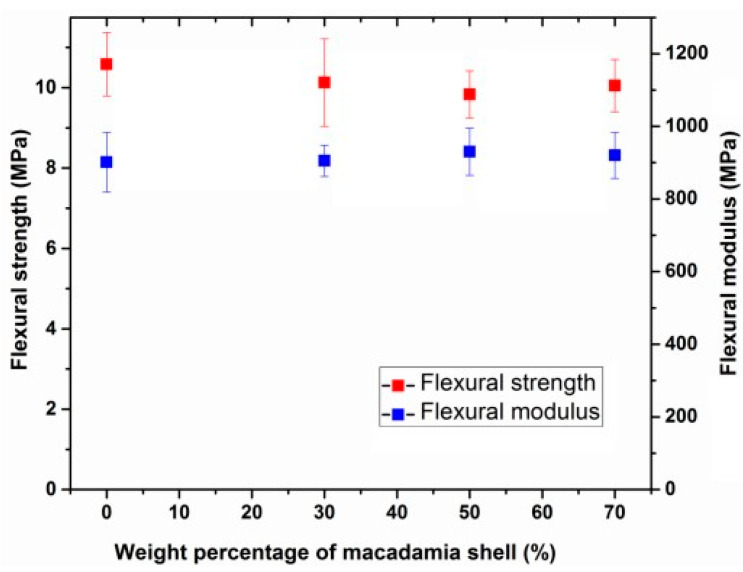
Flexural strength and flexural modulus of WPC panels. Reprinted with permission from Ref. [15]. Copyright 2017, Elsevier.

The comprehensive modulus of the panel board increased as the proportion of macadamia shells increased, reaching a value of 548 MPa for a board containing a 75% macadamia shell mixture. However, in the flexural test, the incorporation of macadamia shell did not exhibit a reinforcing effect and led to a slight decrease in flexural strength (Figure 16). The report suggests that this result may be attributed to the poor interaction between the automotive waste plastics and macadamia shells or the immiscibility of the plastics. Overall, the study demonstrates that the addition of macadamia shells significantly increased the comprehensive modulus by 548 MPa in a WPC panel containing 75% macadamia shell.

Microstructure analysis of the fractured surfaces under compression revealed that the addition of macadamia shells partially transformed the brittle failure of the waste automotive plastic into a ductile failure. In terms of thermal properties, the WPC panel exhibited favorable flame-retardant properties compared to panels made solely from 100% plastic.

### 7.6. Macadamia Nutshell Fillers Studied for Purposes Other Than Composites

Jun et al. [4] developed carbon composites using macadamia nut shells, phenolic resin, and carbon fibers for their application as solid adsorbents in coal-fired power stations for the post-combustion capture of CO_2_. The newly developed composites exhibited a performance improvement of over 30% compared to that of their previously developed adsorbents. The introduction of phenolic resin resulted in an enhanced efficiency of CO_2_ adsorption. Yingge et al. [38] utilized macadamia nut shells as a precursor to prepare porous carbon material, which was subsequently used in the fabrication of sulfur–carbon composite material as the sulfur storage matrix for lithium–sulfur batteries. The study investigated the effect of temperature on the microstructure and electrochemical performance of the porous carbon material. The activation process at a temperature of 900 °C resulted in the desired pore structure of the carbon material. The material exhibited a super high specific surface area (3552.7 m^2^/g), larger pore volume (2.2 cm^3^/g), and higher mesoporous content (23.85%), providing significant technical advantages. Macadamia biomass was effectively used as carbon resource in cleaner production of iron [18] and materials for additive manufacturing [39].

## 8. Concluding Remarks

The physical characteristics of polymers and cellulosic fibers are influenced by various internal factors, including molecular weight, crystallinity, crystal morphology, crosslinking, branching, copolymerization, plasticization, molecular orientation, and residual stress. Additionally, external variables such as ambient atmosphere, the nature of deformation, thermal history, weathering, time (aging), and the frequency of stressing also play a role. The viscoelastic nature of polymers and their composites further demonstrates a strong dependence of mechanical properties on time and temperature. As a result, understanding the intricate relationships between polymer structure and properties is a complex and ongoing area of research, offering intriguing avenues for exploration.

By controlling the nucleation and growth of mineral phases, as well as manipulating the microstructure in materials such as synthetic polymers and biomaterials, it becomes possible to significantly alter their morphology and unlock advanced properties. This knowledge opens up new possibilities for developing novel processing methods and creating useful objects with enhanced functionalities. The mechanical properties such as strength, etc., of macadamia nutshell fibers are naturally high, which is a prime vantage. To be able to utilize such a naturally grown high strength fiber is a great scientific achievement. Furthermore, the fiber and its composites do not inherit the characteristics, e.g., high moisture absorption, that discourage its uses. In this context, exploring innovative approaches becomes crucial in order to fully harness the potential of natural fibers and fillers, such as macadamia nutshells, in the development of functional and structural composite materials.

## Figures and Tables

**Table 1:** Proximate analysis of macadamia shell. Adapted with permission from Ref. [15]. Copyright 2017, Elsevier.

Proximate Analysis (wt% as Received)		Ultimate Analysis (wt% as Received)	
Moisture	5.5	C	48.39
Ash	0.2	O	40.31
Volatile matter	73.5	N	0.333
Fixed carbon	20.8	—	—

**Table 2:** Elemental analysis (X-ray Fluorescence studies) Adapted with permission from Ref. [15]. Copyright 2017, Elsevier.

Analyte	Na	Mg	Al	Si	P	S	Cl	K
Concentration (%)	0.0298	0.0450	0.0620	0.0770	0.0140	0.0400	0.0008	0.1550
Analyte	Ca	Cr	Mn	Fe	Ni	Co	Cu	Zn
Concentration (%)	0.0350	0.0008	0.0047	0.0113	-	0.0001	0.0015	0.0005
Analyte	Se	Zr	Br	Ba	Rb	Hf	Cd	Pb
Concentration (%)	0.0004	-	0.0004	-	0.0001	-	0.0001	0.0002

**Table 3:** Young’s modulus (E), fracture strength (σ_UTS_), and fracture toughness (K_IC_) of macadamia nutshell (‘green’). Adapted with permission from Ref. [13]. Copyright 1995, Kluwer Academic Publishers.

Position	Equator		North/South Pole	N45 °	S45 °
Direction	Polar	Equatorial	Polar/Equatorial	Equatorial	Equatorial
E (GPa)	7.77 (±1.2)	5.38 (±2.43)	4.78 (±2.9)	5.09	4.4
σ_UTS_ (MPa)	61.0 (±5.98)	57.8 (±9.95)	—	56.1	46.2
K_IC_ (MPa√m)	0.81 (±0.18)	0.64 (±0.09)	0.85 (±0.05)	0.9	0.83

**Table 4:** Effect of heat treatment on mechanical properties. Adapted with permission from Ref. [15]. Copyright 2017, Elsevier.

Heat Treatment	Green	Dried	Boiled
Young’s Modulus (GPa)	6.2	5.66	5.95
Tensile Strength (MPa)	57.8 (±8.7)	54.9 (±3.1)	59 (±7)
Compressive Strength (MPa)	80 (±12)	84 (±9)	76 (±8)
Fracture Toughness (MPa√m)	0.78 (±0.12)	0.77 (±0.1)	0.80 (±0.15)
Work of Fracture (Jm^−2^)	98	105	108

## Data Availability

Not applicable.
